# Supplementary figures and images for: New Andean source of resistance to anthracnose and angular leaf spot: Fine-mapping of disease-resistance genes in California Dark Red Kidney common bean cultivar
Source: PLoS One. 2020 Jun 29;15(6):e0235215. doi: 10.1371/journal.pone.0235215 (PMC7323968; doi:10.1371/journal.pone.0235215)

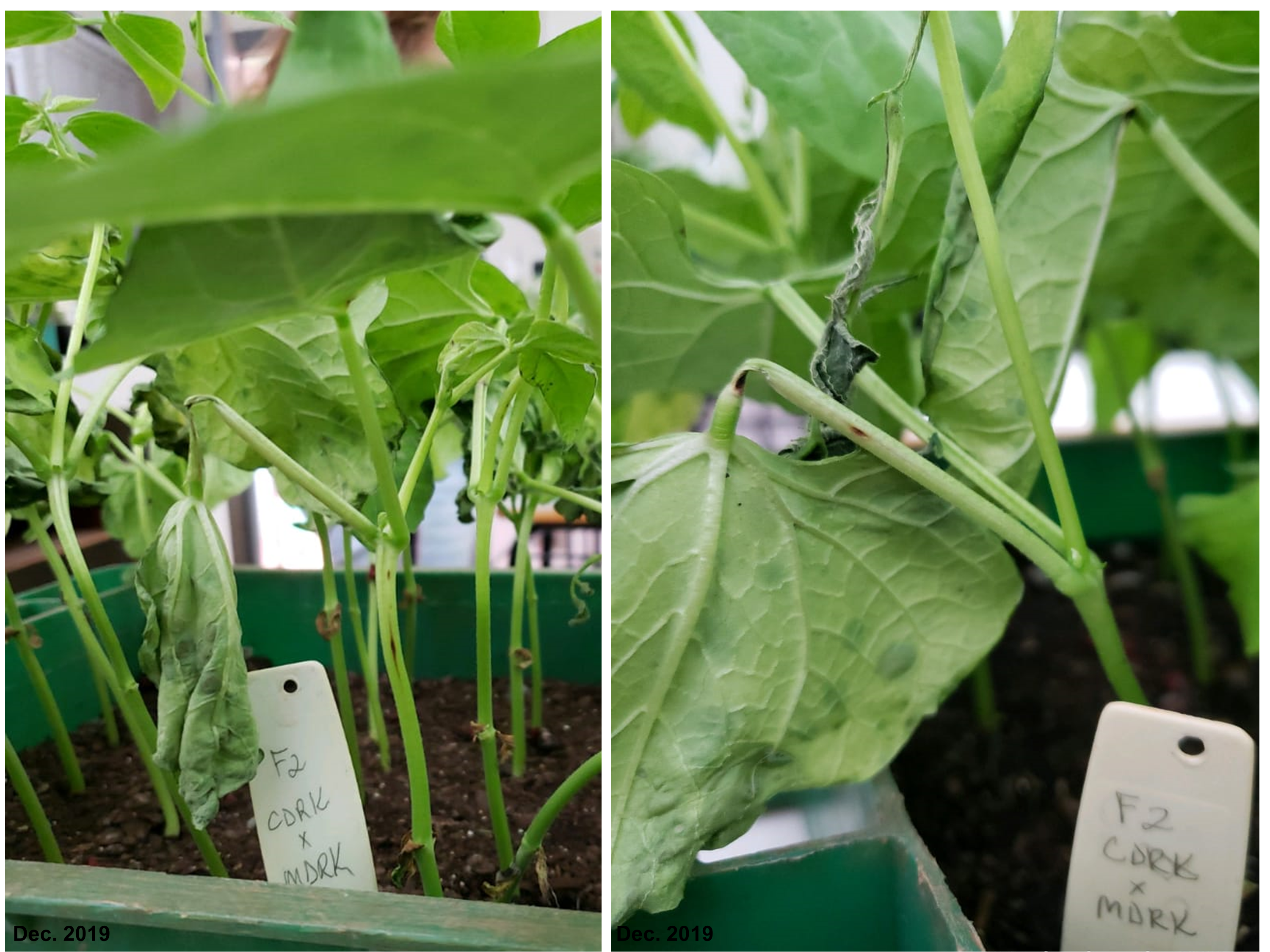

Supplement: S1 Fig — (TIF) [file pone.0235215.s001.tif]
