# Supplementary material for: New Andean source of resistance to anthracnose and angular leaf spot: Fine-mapping of disease-resistance genes in California Dark Red Kidney common bean cultivar
Source: PLoS One. 2020 Jun 29;15(6):e0235215. doi: 10.1371/journal.pone.0235215 (PMC7323968; doi:10.1371/journal.pone.0235215)
Supplement: S1 Table — Ten plants per each RIL were evaluated. Lines written in italics underlined carry recombinant events in the predicted location of the CoCDRK/PhgCDRK loci. (DOC) [file pone.0235215.s002.doc]

**Table S1.** Disease reaction (resistance = R or susceptibility = S) in 110 F_10_ RILs (CY = California Dark Red Kidney × Yolano population) to races 73, 2047 and 3481 of *C. lindemuthianum* and race 63-39 of *P. griseola.* Ten plants per each RIL were evaluated. Lines written in italics underlined carry recombinant events in the predicted location of the *Co^CDRK^/Phg^CDRK^* locus.

|  | **Pathogen Race** | | | | |  | | **Pathogen Race** | | | | |  | | **Pathogen Race** | | | | |
| --- | --- | --- | --- | --- | --- | --- | --- | --- | --- | --- | --- | --- | --- | --- | --- | --- | --- | --- | --- |
| **Lines** | | **63-39** | **73** | **3481** | **2047** | | **Lines** | | **63-39** | **73** | **3481** | **2047** | | **Lines** | | **63-39** | **73** | **3481** | **2047** |
| CY 1 | | S | S | S | S | | CY 50 | | S | S | S | S | | CY 94 | | S | S | S | S |
| CY 2 | | S | S | S | S | | CY 51 | | S | S | S | S | | CY 95 | | R | R | R | R |
| CY 3 | | R | R | R | R | | CY 52 | | R | R | R | R | | *CY 96* | | S | S | S | S |
| CY 4 | | R | R | R | R | | CY 53 | | S | S | S | S | | CY 97 | | S | S | S | S |
| *CY 5* | | S | S | S | S | | CY 54 | | R | R | R | R | | CY 98 | | R | R | R | R |
| CY 6 | | R | R | R | R | | CY 55 | | R | R | R | R | | CY 100 | | R | R | R | R |
| CY 7 | | S | S | S | S | | CY 57 | | R | R | R | R | | CY 101 | | R | R | R | R |
| CY 8 | | R | R | R | R | | CY 58 | | R | R | R | R | | CY 102 | | R | R | R | R |
| CY 10 | | R | R | R | R | | CY 59 | | R | R | R | R | | CY 107 | | R | R | R | R |
| CY 11 | | R | R | R | R | | CY 60 | | R | R | R | R | | CY 109 | | S | S | S | S |
| *CY 12* | | R | R | R | R | | *CY 62* | | S | S | S | S | | CY 111 | | R | R | R | R |
| CY 13 | | S | S | S | S | | CY 63 | | R | R | R | R | | CY 112 | | S | S | S | S |
| CY 14 | | S | S | S | S | | CY 66 | | S | S | S | S | | CY 114 | | R | R | R | R |
| CY 16 | | R | R | R | R | | CY 69 | | S | S | S | S | | *CY 115* | | S | S | S | S |
| CY 18 | | S | S | S | S | | *CY 70* | | R | R | R | R | | CY 116 | | R | R | R | R |
| *CY 19* | | S | S | S | S | | CY 71 | | S | S | S | S | | CY 118 | | S | S | S | S |
| *CY 20* | | S | S | S | S | | *CY 73* | | R | R | R | R | | CY 119 | | S | S | S | S |
| CY 24 | | S | S | S | S | | CY 74 | | S | S | S | S | | CY 120 | | S | S | S | S |
| CY 25 | | S | S | S | S | | CY 75 | | S | S | S | S | | CY 121 | | R | R | R | R |
| CY 26 | | S | S | S | S | | CY 76 | | R | R | R | R | | CY 122 | | S | S | S | S |
| CY 28 | | S | S | S | S | | CY 77 | | R | R | R | R | | CY 124 | | S | S | S | S |
| CY 29 | | R | R | R | R | | CY 78 | | R | R | R | R | | CY 126 | | R | R | R | R |
| CY 30 | | R | R | R | R | | *CY 79* | | S | S | S | S | | CY 127 | | S | S | S | S |
| CY 32 | | R | R | R | R | | CY 80 | | R | R | R | R | | CY 134 | | R | R | R | R |
| *CY 33* | | R | R | R | R | | CY 81 | | S | S | S | S | | CY 136 | | R | R | R | R |
| CY 34 | | S | S | S | S | | CY 82 | | S | S | S | S | | CY 138 | | S | S | S | S |
| CY 35 | | R | R | R | R | | CY 83 | | S | S | S | S | | CY 143 | | S | S | S | S |
| CY 36 | | R | R | R | R | | CY 84 | | S | S | S | S | | CY 144 | | R | R | R | R |
| CY 37 | | S | S | S | S | | CY 85 | | S | S | S | S | | *CY 146* | | R | R | R | R |
| *CY 38* | | S | S | S | S | | CY 86 | | S | S | S | S | | CY 148 | | S | S | S | S |
| CY 39 | | S | S | S | S | | *CY 87* | | S | S | S | S | | CY 152 | | S | S | S | S |
| *CY 43* | | R | R | R | R | | *CY 88* | | S | S | S | S | | CY 153 | | R | R | R | R |
| CY 44 | | S | S | S | S | | CY 89 | | R | R | R | R | | CY 154 | | R | R | R | R |
| CY 45 | | S | S | S | S | | CY 90 | | S | S | S | S | | CY 155 | | S | S | S | S |
| CY 46 | | R | R | R | R | | *CY 91* | | R | R | R | R | | CY 157 | | R | R | R | R |
| *CY 47* | | R | R | R | R | | CY 92 | | S | S | S | S | | CY 159 | | R | R | R | R |
| *CY 48* | | R | R | R | R | | CY 93 | | R | R | R | R | |  | |  |  |  |  |
