# Supplementary material for: New Andean source of resistance to anthracnose and angular leaf spot: Fine-mapping of disease-resistance genes in California Dark Red Kidney common bean cultivar
Source: PLoS One. 2020 Jun 29;15(6):e0235215. doi: 10.1371/journal.pone.0235215 (PMC7323968; doi:10.1371/journal.pone.0235215)
Supplement: S2 Table — Gene positions and their functional annotations are based on the bean reference genome available at Phytozome. (DOC) [file pone.0235215.s003.doc]

**Table S2.** Gene models found in the Pv01 region between 50,301,592 and 50,301,592 delimited by the markers ss715645251 and ss715645248, respectively. Gene positions and their functional annotations are based on the bean reference genome available at Phytozome.

| **Gene Model** | **Gene position** | | | | **Coding sequence length (bp)** | **Functional annotation** |
| --- | --- | --- | --- | --- | --- | --- |
| Phvul.001G243800 | 50,300,459 | 50,303,474 | | | 2421 | Serine/Threonine-protein kinase-like protein CCR3-related |
| Phvul.001G243900 | 50,331,097 | 50,336,473 | | | 3,240 | Double CLP-N motif-containing P-LOOP nucleoside triphosphate hydrolases superfamily protein |
| Phvul.001G244000 | 50,347,010 | 50,350,455 | | | 1,083 | Leucocyanidin oxygenase / Leucoanthocyanidin dioxygenase |
| Phvul.001G244100 | 50,355,553 | 50,358,623 | | | 825 | Phosphoribosylanthranilate isomerase / PRAI |
| Phvul.001G244200 | 50,360,818 | 50,363,438 | | | 651 | Large subunit ribosomal protein L10Ae (RP-L10Ae, RPL10A) |
| Phvul.001G244300 | 50,365,442 | 50,377,656 | | | 5,106 | Clathrin heavy chain (CLTC) |
| Phvul.001G244400 | 50,378,875 | 50,381,218 | | | 753 | Family not named |
| Phvul.001G244500 | 50,385,367 | 50,387,821 | | | 819 | Sterol regulatory element-binding protein // subfamily not named |
| Phvul.001G244600 | 50,397,046 | 50,398,534 | | | 525 | RING-H2 finger protein ATL66 |
| Phvul.001G244700 | 50,407,242 | 50,408,649 | | | 528 | Lob domain-containing protein 21 |
| Phvul.001G244800 | 50,417,584 | 50,419,586 | | | 1,437 | COA-dependent acyltransferase-related |
| Phvul.001G244900 | 50,426,202 | 50,428,382 | | | 600 | UDP-glucose pyrophosphorylase 3 |
| Phvul.001G245000 | 50,435,188 | 50,439,843 | | | 1,605 | Outer membrane protein insertion porin family (SAM50, TOB55, bamA) |
| Phvul.001G245100 | 50,441,273 | 50,443,822 | | | 1,557 | Mitotic checkpoint serine/threonine-protein kinase bub1 // subfamily not named |
| Phvul.001G245200 | 50,444,014 | 50,444,541 | | | 528 | Pollen proteins Ole e I like (Pollen_Ole_e_I) |
| Phvul.001G245300 | 50,447,480 | 50,452,182 | | | 1,932 | Protein tyrosine kinase (Pkinase_Tyr) // Leucine rich repeat N-terminal domain (LRRNT_2) |
| Phvul.001G245400 | 50,462,536 | 50,463,243 | | | 708 | Glutaredoxin // subfamily not named |
| Phvul.001G245500 | 50,466,278 | 50,472,041 | | | 1,785 | Methyltransferase pmt22-related |
| Phvul.001G245600 | 50,474,458 | 50,477,082 | | | 1,023 | translation initiation factor 2 subunit 1 (EIF2S1) |
| Phvul.001G245700 | 50,478,825 | 50,481,129 | | | 795 | Ferritin heavy chain (FTH1) |
| Phvul.001G245800 | 50,490,786 | 50,491,694 | | | 546 | Lob domain-containing protein 12 |
| Phvul.001G245900 | 50,502,661 | 50,510,131 | | | 2,202 | Mannosyl-glycoprotein endo-beta-N-acetylglucosaminidase / Endo-beta-N-acetylglucosaminidase |
| Phvul.001G246000 | 50,512,380 | 50,517,494 | | | 1,767 | ATP-dependent RNA helicase DDX55/SPB4 [EC:3.6.4.13] (DDX55, SPB4) |
| Phvul.001G246100 | 50,519,082 | 50,526,230 | | | 873 | Cation-dependent mannose-6-phosphate receptor |
| Phvul.001G246200 | 50,528,582 | 50,532,938 | | | 1,260 | Protein trichome birefringence-like 33 |
| Phvul.001G246300 | 50,540,616 | 50,541,930 | | | 621 | Abscisic acid receptor PYL5 |
| Phvul.001G246400 | 50,545,702 | 50,550,616 | | | 3,537 | SNF2 domain-containing protein classy 1-related |
| Phvul.001G246800 | 50,572,698 | | 50,575,762 | 2,556 | | Leucine-rich repeat receptor-like protein kinase imk3-related |
