# Supplementary material for: New Andean source of resistance to anthracnose and angular leaf spot: Fine-mapping of disease-resistance genes in California Dark Red Kidney common bean cultivar
Source: PLoS One. 2020 Jun 29;15(6):e0235215. doi: 10.1371/journal.pone.0235215 (PMC7323968; doi:10.1371/journal.pone.0235215)
Supplement: S3 Table — *Genetic position in centiMorgans (cM), based on the map developed by Song et al. (2015). (DOC) [file pone.0235215.s004.doc]

**Table S3.** SNP markers associated with the anthracnose resistance locus in the common bean cultivar CDRK discovered by co-segregation and genetic mapping analysis and located on the lower end of chromosome Pv01 of common bean. *Genetic position in centiMorgans (cM), based on the map developed by Song et al. (2015).

| NCBI ID | BARCBEAN6K_3 SNP id | Genetic position (cM)* | SNP physical |
| --- | --- | --- | --- |
| Code |  |  | Position |
| ss715646585 | sc00076ln674865_383201_A_G_75705477 | 58.09 | 48,448,199 |
| ss715645886 | sc00022ln1003704_463708_T_C_32965580 | - | 49,303,661 |
| ss715645859 | sc00022ln1003704_178718_A_G_32680590 | 63.93 | 49,588,715 |
| ss715645856 | sc00022ln1003704_150441_G_A_32652313 | 63.93 | 49,617,274 |
| ss715645853 | sc00022ln1003704_129339_C_A_32631211 | 63.93 | 49,637,944 |
| ss715645852 | sc00022ln1003704_109623_C_T_32611495 | 63.93 | 49,657,760 |
| ss715645935 | sc00022ln1003704_96352_G_A_32598224 | 64.12 | 49,671,031 |
| ss715645891 | sc00022ln1003704_49856_T_G_32551728 | 64.64 | 49,718,129 |
| ss715645862 | sc00022ln1003704_22533_G_A_32524405 | 64.64 | 49,742,126 |
| ss715645855 | sc00022ln1003704_14515_T_C_32516387 | 64.65 | 49,749,711 |
| ss715645288 | sc00003ln2130026_2113635_G_A_6438335 | 64.86 | 49,783,658 |
| ss715645287 | sc00003ln2130026_2105132_A_G_6429832 | 65.16 | 49,793,139 |
| ss715645286 | sc00003ln2130026_2057851_G_A_6382551 | - | 49,841,858 |
| ss715645284 | sc00003ln2130026_2037421_C_A_6362121 | - | 49,862,290 |
| ss715645280 | sc00003ln2130026_2002999_C_T_6327699 | 65.99 | 49,895,862 |
| ss715645274 | sc00003ln2130026_1923031_T_G_6247731 | 66.59 | 49,969,810 |
| ss715645269 | sc00003ln2130026_1850170_T_C_6174870 | 66.79 | 50,042,771 |
| ss715645266 | sc00003ln2130026_1827265_G_A_6151965 | 66.79 | 50,065,488 |
| ss715645263 | sc00003ln2130026_1796976_T_C_6121676 | 66.79 | 50,093,966 |
| ss715645262 | sc00003ln2130026_1791042_A_G_6115742 | 66.79 | 50,099,818 |
| ss715645260 | sc00003ln2130026_1773813_T_C_6098513 | 66.79 | 50,115,685 |
| ss715645258 | sc00003ln2130026_1733138_T_C_6057838 | 66.99 | 50,155,987 |
| ss715645257 | sc00003ln2130026_1727598_G_T_6052298 | 66.99 | 50,161,526 |
| ss715645256 | sc00003ln2130026_1706192_A_C_6030892 | 66.99 | 50,182,775 |
| ss715645254 | sc00003ln2130026_1685328_C_T_6010028 | 66.99 | 50,203,547 |
| ss715645252 | sc00003ln2130026_1665755_G_T_5990455 | 67.2 | 50,222,584 |
| ss715645251 | sc00003ln2130026_1591193_A_G_5915893 | 67.61 | 50,301,592 |
| ss715645248 | sc00003ln2130026_1344154_T_C_5668854 | - | 50,546,985 |
