# Supplementary material for: New Andean source of resistance to anthracnose and angular leaf spot: Fine-mapping of disease-resistance genes in California Dark Red Kidney common bean cultivar
Source: PLoS One. 2020 Jun 29;15(6):e0235215. doi: 10.1371/journal.pone.0235215 (PMC7323968; doi:10.1371/journal.pone.0235215)
Supplement: S4 Table — (DOC) [file pone.0235215.s005.doc]

**Table S4.** Allelism tests in F_2_ populations for the anthracnose resistance gene in the common bean cultivar California Dark Red Kidney (CDRK).

| Crosses | Resistance Gene | Race | Linkage Group | Observed Ratio | | Expected Ratio (R:S) | χ^2^ | *P-value* |
| --- | --- | --- | --- | --- | --- | --- | --- | --- |
|  |  |  |  | R^c^ | S^d^ |  |  |  |
| CDRK × MDRK^a^ | *Co-1* | 3481 | Pv01 | 280 | 18 | 15:1 | 0.022 | 0.88 |
| CDRK × AC^b^ | *Co-AC* | 3481 | Pv01 | 113 | 8 | 15:1 | 0.026 | 0.87 |

MDRK^a^ = Michigan Dark Red Kidney; AC^b^ = Amendoim Cavalo; R^c^ = Resistant; S^d^ = Susceptible
